# KNOWLEDGE OF LGBT CULTURAL SENSITIVITY AMONG AGING SERVICE PROVIDER NETWORKS

**DOI:** 10.1093/geroni/igz038.2738

**Published:** 2019-11-08

**Authors:** Austin Oswald, Daniel S Gardner, Tim R Johnston, Sabretta G Alford, Nancy Giunta

**Affiliations:** 1 The Graduate Center, CUNY, New York, New York, United States; 2 Silberman School of social Work, Hunter College, CUNY, New York, New York, United States; 3 SAGE, Los Angeles, California, United States

## Abstract

The population of lesbian, gay, bisexual, and transgender (LGBT) adults aged 50 or above is rapidly aging, posing distinct challenges to aging service providers who work to meet their complex health and social needs. In 2011, Services and Advocacy for GLBT Elders (SAGE), home of the National Resource Center on LGBT Aging (NRC), developed and implemented a suite of trainings designed to sensitize aging service providers to the needs and experiences of LGBT elders. This descriptive study examines baseline (pre-test) survey data from individuals trained between 2013 and 2018 (N=10,500). Training participants represent all states throughout the U.S. and vary widely in race/ethnicity, age, and job title. Preliminary results indicate that knowledge measured prior to trainings shows substantial variation across geographic regions, suggesting the need for more geographically targeted approaches to training delivery. Implications for practice, research, and policy with LGBT adults in later life will be discussed.

